# The association between palatal rugae pattern and dental malocclusion

**DOI:** 10.1590/2177-6709.24.1.37.e1-9.onl

**Published:** 2019

**Authors:** Farheen Fatima, Mubassar Fida, Attiya Shaikh

**Affiliations:** 1 The Aga Khan University Hospital, Section of Dentistry, Department of Surgery (Karachi, Pakistan).; 2 Liaquat College of Medicine and Dentistry, Department of Orthodontics (Karachi, Pakistan).

**Keywords:** Palate, Malocclusion, Dental models

## Abstract

**Introduction:**

Palatal rugae complete its development during early intrauterine life, whereas dental malocclusions in permanent dentition establishes several years into the post-natal life.

**Objective:**

The objective of present study was to determine if there is an association between the palatal rugae pattern and Angle’s classes of malocclusion.

**Methods:**

A cross-sectional study was conducted on pretreatment dental casts of 184 patients. The sample was divided into the following groups: Class I, Class II div. 1, Class II div. 2, and Class III. The number of palatal rugae was recorded, bilaterally. The length, pattern and orientation of three anterior-most primary rugae on both sides were recorded.

**Results:**

The mean age of the study sample was 17.8 ± 5.4 years. The mean number of the palatal rugae was 11.18 ± 2.5, with significant differences among different malocclusion groups. The length of the first rugae on left side and third rugae on both sides varied significantly among the groups (*p*< 0.05). Similarly, the pattern of palatal rugae was also found to be significantly different among the malocclusion groups. The right sided rugae did not have any significant difference in the orientation in different malocclusion groups; however, the left sided rugae showed significant differences among the four malocclusion groups (*p*< 0.001).

**Conclusions:**

The current study showed subtle differences in the palatal rugae pattern among the Angle’s classes of malocclusion. Similarly, the length and orientation of some rugae were also found to be significantly different between malocclusion groups.

## RESUMO

**Introdução:**

as rugas palatinas completam seu desenvolvimento ainda no início da vida intrauterina, enquanto as más oclusões dentárias na dentição permanente se estabelecem após vários anos de vida pós-natal.

**Objetivos:**

o presente estudo foi planejado para determinar se há associação entre o padrão das rugas palatinas e as más oclusões, segundo a classificação Angle.

**Métodos:**

um estudo transversal foi conduzido a partir dos modelos dentários pré-tratamento de 184 pacientes. A amostra foi dividida em quatro grupos: Classe I; Classe II, divisão 1; Classe II, divisão 2 e Classe III. O número de rugas palatinas foi registrado bilateralmente. O comprimento, padrão e a orientação das três rugas primárias mais anteriores de ambos os lados foram registrados.

**Resultados:**

a idade média da amostra desse estudo foi de 17,8 ± 5,4 anos. O número médio de rugas palatinas foi de 11,18 ± 2,5, com diferenças significativas entre os grupos de más oclusões. O comprimento da primeira ruga do lado esquerdo e da terceira ruga em ambos os lados variou significativamente entre os grupos (*p*< 0,05). De forma semelhante, o padrão das rugas palatinas também mostrou diferença significativa entre os grupos com diferentes má oclusões. As rugas do lado direito não apresentaram nenhuma diferença significativa de orientação nos diferentes grupos; no entanto, as rugas do lado esquerdo apresentaram diferenças significativas nos grupos estudados (*p*< 0,001).

**Conclusões:**

o presente estudo demonstrou diferenças sutis quanto ao padrão das rugas palatinas entre as classes de má oclusão definidas por Angle. Do mesmo modo, o comprimento e a orientação de algumas rugas também se apresentaram consideravelmente diferentes entre os grupos com diferentes má oclusões.

## INTRODUCTION

The palatal rugae are unique structures that are inalterable in their position and pattern throughout the life of an individual. This imparts them a special role in the forensic Dentistry, having potential implications in the process of human identification.^1^ ^-^ ^3^ In Orthodontics, they are used as stable reference landmarks for the superimposition of pre- and post-treatment cephalometric tracings.^4^ Palatal rugae appear during the third month of intrauterine life and occupy most of the length of palatal shelves. These structures form a series of anatomical folds on the anterior part of the palatal mucosa, behind the incisive papilla on each side of the median palatal raphe.^5^ The number varies from 4 to 6 on each side, and they appear before the fusion of the palatine shelves. Their growth and development is controlled by the interaction between epithelial and mesenchymal cells. By the end of intrauterine life, the pattern becomes irregular, the posterior ones disappear and the anterior ones increases in prominence.^6^ ^-^ ^8^ They are protected by the surrounding soft and hard tissues - i.e., lips, cheeks, tongue, teeth and bone -, which guards them against trauma and high temperature. Their role has been established in deglutition and oral sensation; however, their precise role in sensorineural function is not entirely understood.^9^ ^,^ ^10^

The craniofacial growth and development occur via complex interaction between fibroblast growth factors and Hedgehog signaling pathways.^11-13^Some studies have reported strong contribution of genetic factors to the malocclusion susceptibility.^14^ ^,^ ^15^ Polygenic inheritance has been reported for Class II subdivision 1; while Class II subdivision 2 and Class III malocclusion showed autosomal dominant inheritance.^16-18^ In the recent years, several papers have been published exploring the genetic pathways and molecular basis during the formation of palatal rugae.^3^ ^,^ ^11^ ^,^ ^13^ ^,^ ^19^

Malocclusion causes aesthetic concerns and functional impairment that may result in long term impact on the psychological health. An emphasis has been placed on the early diagnosis, as this may provide an advantage of preventive or interceptive treatment that may reduce disease burden and treatment duration.^20^ Since, the palatal rugae are stable structures and follow common signaling pathway during craniofacial development, its association with Angle’s classes of malocclusion can be helpful for the prediction of forthcoming dentoskeletal aberrations. A survey of pertinent literature showed that, apart from a pilot study, no data has been available describing association of palatal rugae with various malocclusions traits.^15^ Therefore, the purpose of the present study was to investigate whether an association exists between morphological features of palatal rugae and Angle’s classes of malocclusion. 

## MATERIAL AND METHODS

A retrospective cross-sectional study was conducted on the pre-treatment dental casts of 184 patients (92 males, 92 females) presenting to treatment in the last five years. Pretreatment dental records of 5,000 subjects were evaluated, and subjects who matched the inclusion criteria were selected. Ethical clearance was obtained from the ethical review committee (reference #4075-16) prior to data collection. The sample size was calculated using the findings of Gandikota et al,^21^ who reported mean length of primary palatal rugae as 20.54 ± 2.46 mm and 19.11 ± 1.78 mm in Angle’s Class I and Class II, respectively. The power was set at 80% and alpha was kept as 0.05.

### Subjects and study groups

The sample was divided into four equal groups, with 46 subjects in each group. The subjects were categorized on the basis of molar and incisor relationships (i.e. Class I, Class II division 1, Class II division 2 and Class III), and full unit cases were considered respective to incisor relations. Each malocclusion group had equal number of male and female subjects.

The sample was obtained from Pakistani population as confirmed by National identity card. Only subjects with good-quality dental casts were included in the study. All subjects were in the age range of 12-30 years, with full permanent dentition, well-established molar and incisor relationships and normal vertical growth pattern. Subjects with quarter or half-cusp molar relation, subdivision and asymmetric cases, and complex cases with unmatched molar and incisor relations were excluded. Moreover, patients with history of extraction or previous orthodontic treatment, cleft lip and palate, craniofacial and dental anomalies, pathology or trauma involving the head and neck region, habits such as tongue thrusting or thumb sucking and carious or missing molars and incisors were also excluded from the study.

### Dental cast analysis

**Figure 1:**
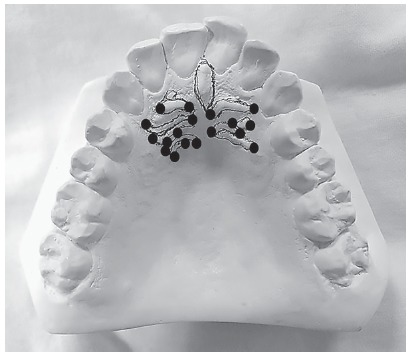
Palatal rugae tracing on dental cast.

Study was conducted on the high-quality pre-treatment dental stone models (white orthodontic stone, ISO type 3) derived from the alginate impressions of upper and lower dental arches. The palatal rugae were outlined with a sharp HB pencil under suitable light, and magnification. The most medial and distal ends of the palatal rugae were marked on dental cast and linear distances were measured using digital vernier calipers (0-150 mm ME00183, Dentaurum, Pforzheim, Germany) (Fig 1). 

### Assessment of features of palatal rugae

**Figure 2:**
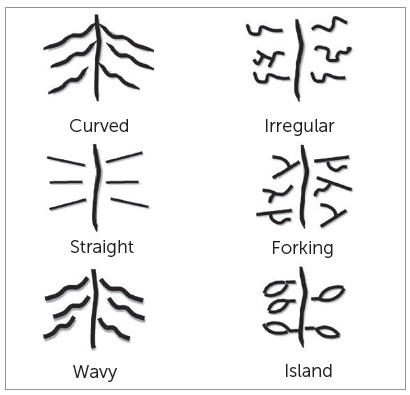
Patterns of palatal rugae.

**Figure 3:**
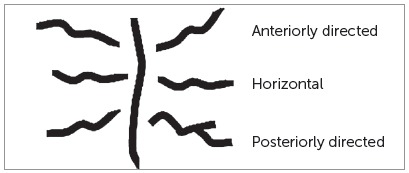
Orientation of palatal rugae.

Based on length, the rugae were categorized as primary (> 5 mm), secondary (3 - 5 mm), and fragmentary type (< 3 mm).^8^ The total number of rugae was recorded for both right and left sides. The three anterior-most primary rugae (labelled as ruga 1, 2 and 3) were observed for the length, pattern and orientation. For the assessment of pattern and orientation, the rugae were classified according to the method described by Hauser et al ^5^ (Figs 2 and 3).

## STATISTICAL ANALYSIS

Data were analyzed using SPSS for Windows (version 20.0, SPSS Inc. Chicago). The Shapiro-Wilk test was applied to test the normality of data, which showed a non-normal distribution; hence non-parametric test was applied. The Mann-Whitney U test was applied to compare the study parameters between genders. Descriptive statistics for the palatal rugae lengths, i.e. means and standard deviations (SD), were calculated. The Kruskal-Wallis test was used to compare the mean differences in the palatal rugae number and length among the four malocclusion groups. The pattern and orientation were compared across the four groups using the Chi-square test. To rule out any error in measurement, 30 dental casts were reevaluated by the main investigator using intraclass correlation coefficient for quantitative variables and Kappa statistics for the qualitative variables. A p-value ≤ 0.05 was considered as statistically significant.

## RESULTS

The measurement error was evaluated and the results showed fair to excellent agreement between the two sets of reading in the assessment of rugae number and length, whereas pattern and orientation assessment showed moderate to excellent agreement (Tables 1 and 2). The comparison between sexes, regarding number of palatal rugae, and length, pattern and orientation of primary rugae, showed insignificant differences; therefore, further statistical analyses were not performed separately. 

**Table 1:** Assessment of the reliability of measurements.

Palatal rugae			Mean ± SD		ICC
			First reading (n = 30)	Second reading (n = 30)	
Number of rugae	Primary	Right	4.27 ± 9.8	4.13± 1.1	0.9
		Left	4.50 ± 1.1	4.63 ± 1.2	0.8
	Secondary	Right	1.27 ± 9.4	1.60 ± 1.0	0.8
		Left	1.13 ± 1.0	1.33 ± 0.9	0.8
	Fragmentary	Right	0.43 ± 0.6	0.93 ± 0.8	0.7
		Left	0.40 ± 0.6	0.80 ±0.7	0.7
Length of primary rugae (mm)	First	Right	8.51 ± 1.2	8.69 ± 1.4	0.9
		Left	8.19± 2.0	8.58 ± 2.0	0.9
	Second	Right	8.44 ± 2.0	8.77 ± 2.0	0.9
		Left	8.82 ± 1.9	8.99 ± 2.3	0.9
	Third	Right	10.14 ± 2.5	10.06 ± 2.5	0.9
		Left	9.17 ± 2.5	9.31 ± 2.4	0.9

n = 30; SD = standard deviation; > 0.75 = excellent agreement; 0.4 - 0.75 = fair agreement; < 0.4 = poor agreement.

ICC = Intraclass correlation coefficient.

Statistically significant differences were found in the number of rugae among malocclusion groups (Table 3). The mean lengths of the primary rugae are shown in Table 4. There were significant differences in mean lengths among the groups in ruga 1 on left side (*p*< 0.001) and rugae 3 on both right (*p*= 0.03) and left (*p*< 0.001) sides. Curved pattern was predominant and significant differences were found among the groups (*p*< 0.05); however, the results did not show any specific pattern peculiar to any malocclusion type. The distribution of different rugae pattern is shown in Table 5. There was no significant differences for the orientation among the groups on right side; however, left side showed significant differences for the ruga 1 (*p*= 0.001), ruga 2 (*p*= 0.004) and ruga 3 (*p*= 0.013). The distribution of orientation of the rugae among the malocclusion groups is shown in Table 6.

**Table 2:** Assessment of the reliability of measurements.

Palatal rugae			κ	p-value
Pattern of primary rugae	First	Right	0.6	0.00
		Left	0.8	0.00
	Second	Right	0.6	0.00
		Left	0.8	0.00
	Third	Right	0.6	0.00
		Left	0.6	0.00
Orientation of primary rugae	First	Right	0.6	0.00
		Left	0.6	0.00
	Second	Right	0.7	0.00
		Left	0.8	0.00
	Third	Right	0.9	0.00
		Left	0.8	0.00

n = 30; SD = standard deviation; κ = Cohen’s kappa; ≤ 0 = no agreement; 0.01-0.20 = none to slight; 0.21-0.40 = fair; 0.41- 0.60 = moderate; 0.61-0.80 = substantial; 0.81-1.00 = perfect agreement.

**Table 3:** Mean number of palatal rugae among malocclusion groups.

Number of rugae			Molar Class			p-value
			Class I	Class II/1	Class II/2	Class III
Primary	Right	4.33 ± 1.0	3.87 ± 1.0	3.87 ± 0.8	4.02 ± 0.7	0.05*
	Left	4.37 ± 1.0	3.70 ± 0.6	4.04 ± 0.8	4.07 ± 0.8	< 0.001**
Secondary	Right	1.07 ± 0.9	1.26 ± 0.8	0.70 ± 0.8	1.00 ± 0.8	< 0.001**
	Left	1.22 ± 0.9	1.24 ± 0.7	0.83 ± 0.8	0.89 ± 0.8	0.02*
Fragmentary	Right	0.28 ± 0.5	0.41 ± 0.7	0.54 ± 0.9	0.67 ± 0.7	0.01*
	Left	0.35 ± 0.6	0.59 ± 0.6	0.76 ± 0.7	0.67 ± 0.8	0.02*

n = 184; Kruskal-Wallis test, *p < 0.05; ** p < 0.001.

**Table 4:** Mean lengths of palatal rugae among malocclusion groups.

Length of primary rugae (mm)			Molar Class			p-value
			Class I	Class II/1	Class II/2	Class III
First	Right	8.64 ± 1.3	8.27 ± 1.5	8.16 ± 1.2	8.56 ± 1.2	0.36
	Left	8.34 ±1.9	9.60 ± 2.0	9.62 ± 1.1	9.01 ± 1.2	<0.001**
Second	Right	8.95 ± 2.5	9.22 ± 2.1	9.25 ± 2.1	9.71 ± 2.4	0.52
	Left	8.59 ± 1.8	8.66 ± 1.7	8.94 ± 2.2	9.69 ± 2.4	0.09
Third	Right	9.70 ± 2.5	10.31 ± 3.1	11.03 ± 2.6	10.87 ± 2.4	0.03*
	Left	9.38 ± 2.1	10.88 ± 2.5	10.60 ± 1.8	11.94 ± 2.3	<0.001*

n = 184; Kruskal-Wallis test, *p < 0.05; ** p < 0.001.

**Table 5:** Comparison of pattern of primary palatal rugae among malocclusion groups.

Patterns of primary rugae			Molar Class			p-value	
			Class I	Class II/1	Class II/2	Class III	
First	Right	Curved	24	26	25	22	0.008*
		Straight	14	6	5	12	
		Wavy	1	0	0	0	
		Irregular	0	0	0	0	
		Forking	7	14	16	8	
		Island	0	0	0	4	
	Left	Curved	26	26	22	14	0.007*
		Straight	11	5	5	12	
		Wavy	0	0	0	0	
		Irregular	0	0	0	0	
		Forking	8	15	19	16	
		Island	1	0	0	4	
Second	Right	Curved	42	20	24	21	<0.001**
		Straight	4	8	8	6	
		Wavy	0	2	7	6	
		Irregular	0	0	0	0	
		Forking	0	16	7	13	
		Island	0	0	0	0	
	Left	Curved	28	21	34	30	0.001**
		Straight	10	7	4	6	
		Wavy	8	4	4	6	
		Irregular	0	0	0	0	
		Forking	0	14	4	4	
		Island	0	0	0	0	
Third	Right	Curved	33	34	36	28	0.003*
		Straight	8	3	0	4	
		Wavy	5	2	5	2	
		Irregular	0	0	0	0	
		Forking	0	7	5	12	
		Island	0	0	0	0	
	Left	Curved	25	38	36	32	0.01*
		Straight	8	1	5	6	
		Wavy	12	2	5	6	
		Irregular	0	0	0	0	
		Forking	1	4	0	2	
		Island	0	0	0	0	

n = 184; Chi-square test, *p < 0.05; ** p < 0.001.

**Table 6:** Comparison of orientation of primary palatal rugae among malocclusion groups.

Orientation of primary rugae			Molar Class			p-value	
			Class I	Class II/1	Class II/2	Class III	
First	Right	Posteriorly directed	26	25	37	31	0.13
		Horizontal	7	9	5	5	
		Anteriorly directed	13	12	4	10	
	Left	Posteriorly directed	24	29	41	37	0.001**
		Horizontal	14	7	2	6	
		Anteriorly directed	8	10	3	3	
Second	Right	Posteriorly directed	7	17	15	16	0.11
		Horizontal	9	5	7	2	
		Anteriorly directed	30	24	24	28	
	Left	Posteriorly directed	20	18	32	31	0.004*
		Horizontal	10	5	6	6	
		Anteriorly directed	16	23	8	9	
Third	Right	Posteriorly directed	9	14	20	10	0.15
		Horizontal	2	1	0	2	
		Anteriorly directed	35	31	26	34	
	Left	Posteriorly directed	14	18	25	29	0.013*
		Horizontal	8	3	7	3	
		Anteriorly directed	24	25	14	14	

n = 184; Chi-square test, *p < 0.05; ** p < 0.001.

## DISCUSSION

The characteristics of palatal rugae are unique to an individual. Variations may be seen among both sexes; however, the literature review shows conflicting evidence in various populations. Some studies have stated insignificant sexual dimorphism in rugae pattern; whereas other studies have reported significant differences among male, female, and transgender populations.^2^ ^,^ ^22^ ^-^ ^25^ However, in the present study, it was found no significant differences between male and female groups regarding the number of palatal rugae, and length, pattern and orientation of primary palatal rugae. The differences in results could be due to ethnic variation. In the current study, the average number of primary rugae was close to three, which is in concordance with the results of previous studies.^6,26^ The mean number of palatal rugae was observed to be greatest in Class I subjects, and the mean lengths of primary rugae were found to be comparable among the four malocclusion groups. In contrast, Kapoor et al^6^ reported the highest number of palatal rugae in Class II division 2 group and shorter length of first, second and third rugae in Class II division 1, as compared to Class I group, in an Indian population. However they conducted a pilot study and the subjects were not evenly distributed in the malocclusion groups, whereas the present study was conducted on an adequate sample with comparable number of subjects in each group.

The role of palatal rugae in mastication, deglutition and speech has been reported in literature.^9^ ^,^ ^10^ Lysell^8^ reported that the dorsal surface of tongue is an important determinant of rugae pattern. Tongue position may vary with type of malocclusion^27^; therefore, the pattern of rugae is expected to vary in different malocclusion classes. In Class II malocclusion, the tip of tongue is positioned more posteriorly, and dorsal portion is postured more superiorly, as compared to skeletal Class I malocclusion.^28^ Moreover, the tongue posture in posterior regions was found to be significantly lower in subjects with Class III malocclusion, as compared to Class I subjects.^27^ The present results showed significant differences in pattern of primary rugae among the study groups. Contrasting results were reported by Kapoor el al,^6^ which could be due to the small sample size in their study, which failed to detect the differences. 

Strong genetic predisposition has been reported in the number, shape and orientation of palatal rugae.^29^ ^,^ ^30^ Orientation of the primary palatal rugae was found to have significant differences on the left side, and the first primary rugae were found to be posteriorly directed more frequently in Class II division 2 group. Previous study reported insignificant differences in the orientation of primary palatal rugae.^7^ The possible reason of conflicting results could be due to the fact that rugae are asymmetric structures and the mechanism of their development and establishment has been poorly understood.^31^ These results suggest that the developments of different structures in orofacial complex are related to each other, and may be subjected to similar epigenetic variations that may influence their phenotype. Further exploration of genetic variations at the molecular level can prove to be the gold standard to establish this relationship.

Different methods for the evaluation of palatal rugae on dental cast have been described in the literature. Optocom software,^32^ Reflex metrograph,^33^ ^,^ ^34^ photographs^5^ and photocopies of dental cast have been used in the past for the evaluation of palatal rugae.^35^ Each of these methods requires a sophisticated instrument, device or software that is not acquired by many investigators and clinicians. Kapali et al^29^ and Moran et al^36^ used slide vernier caliper to measure palatal landmarks. Digital vernier caliper was used in the current investigation as it is user-friendly and can be used directly on dental cast; therefore, does not require cast digitization and particular expertise. 

Limitations of the present study were manual tracing of palatal rugae for the assessment of morphological characteristics, and a single investigator assessing the dental casts. Moreover, assessment of pattern and orientation of rugae is subjective. With recent technological advancement, study casts can be scanned with a three-dimensional scanner and measurements can be done in three dimensions on computer screen, for more reliable results.

## CONCLUSIONS

The current study found an association between the number of palatal rugae and the pattern of primary rugae with the Angle’s classes of malocclusion. However, the length and the orientation of the primary palatal rugae showed variable results. 
